# Comparison of Treatment Outcome of Forsus Fatigue Resistant Device With Single Versus Double Wires: A Randomized Controlled Trial

**DOI:** 10.7759/cureus.103674

**Published:** 2026-02-15

**Authors:** Shweta Turkia, Sharvari Mairal, Vipul Sharma, Jakshmi K J, Ulhaas Kashyap, T P Chaturvedi

**Affiliations:** 1 Orthodontics and Dentofacial Orthopedics, Faculty of Dental Sciences, Institute of Medical Sciences, Banaras Hindu University, Varanasi, IND

**Keywords:** double slot, dual wires, forsus device, single slot, skeletal class ii malocclusion

## Abstract

Background: Skeletal Class II malocclusion with mandibular retrusion as primary etiology is usually managed with treatment strategies such as growth modification, orthodontic camouflage, and surgical correction, depending on the patient’s age and severity of the discrepancy. Patients in the circumpubertal age group are commonly treated with growth-modification appliances. Although removable functional appliances can effectively stimulate mandibular growth, their success is frequently limited by patient compliance and interference with oral function. Fixed functional appliances provide a compliance-independent alternative; however, achieving consistent clinical efficiency and patient comfort remains a challenge. Among these, the Forsus Fatigue Resistant Device (FFRD; 3M Unitek, Monrovia, CA, USA) has emerged as a reliable option, offering continuous orthopedic forces and convenient adjustability for effective Class II correction.

Objective: To evaluate the effectiveness of the FFRD with single versus double wires.

Materials and methods: The study included 24 subjects with skeletal Class II malocclusion, aged 15-21 years. Subjects were randomly assigned to two groups, each consisting of 12 individuals. Group 1 was treated with the Forsus device using single-slot brackets, while Group 2 incorporated the Forsus device with double-slot brackets. Linear and angular parameters were measured on lateral cephalograms before and after Forsus therapy. Statistical analysis included the Shapiro-Wilk, independent t-test, ANOVA, Friedman, and Mann-Whitney U tests.

Results: Class I molar relationship, overjet, and overbite correction were achieved in both the treatment groups. Sagittal correction (p<0.001 and p<0.001), mandibular incisor proclination (p<0.001 and p=0.012), and change in mandibular plane angle (p<0.001 and p=0.002 for groups 1 and 2 respectively) were significant in both the treatment groups. However, no significant differences were found between the treatment groups.

Conclusion: In both treatment groups, correction of Class II malocclusion was primarily dentoalveolar in nature. The use of double wires in double-slot brackets with the Forsus device did not result in statistically significant enhancement of mandibular growth, nor did it mitigate the labial inclination of mandibular incisors.

## Introduction

Class II malocclusion is a common dentofacial discrepancy, with a prevalence ranging from 15% to 30% of the population. It is further subclassified into Division 1 and Division 2, based on maxillary incisor inclination. Class II Division 1 is characterized by excessive labial inclination of maxillary incisors, increased overjet, and with or without a relatively narrowed maxillary arch [1,2]. A characteristic feature is usually the presence of proclined mandibular incisors as a compensatory mechanism to address the overjet. In contrast, Class II Division 2 is characterized by excessive lingual inclination of maxillary incisors, usually accompanied by a deep overbite and minimal overjet. Management strategies for Class II Division 1 correction encompass growth modulation techniques, camouflage approaches, and surgical interventions [3]. Removable functional appliances, while designed to guide mandibular growth, exhibit significant limitations in terms of patient compliance and compromised oral functions [4]. However, while a variety of fixed functional appliances (FFAs) have been utilized in clinical practice, achieving both high patient acceptance and clinically satisfactory outcomes has proven to be an ongoing challenge [5,6]. One of the hybrid FFAs, the Forsus Fatigue Resistant Device (FFRD; 3M Unitek, Monrovia, CA, USA), is easily adjustable, exerting continuous orthodontic forces and offering a versatile option for mandibular advancement [7,8]. While 0.019x0.025-inch wires are generally preferred over the less rigid 0.018x0.025-inch wires for effective appliance function, it's crucial to acknowledge that even these wires can lead to unwanted dentoalveolar changes. Consequently, the persistent challenge of controlling lower incisor inclination can be a barrier to achieving complete skeletal correction. To address these limitations, strategies like the use of skeletal anchorage and increased lingual crown torque in mandibular brackets and wires have been employed to enhance treatment outcomes [1,9-11]. Prior research on fixed functional appliances has utilized single-slot brackets. The introduction of double-slot brackets in 2018 offers new possibilities for optimizing biomechanics in orthodontic treatment [12]. It may be hypothesized that double wires may provide added anchorage for better control of incisors, mitigating undesirable dentoalveolar effects and maximizing skeletal effects. Studies comparing the FFRD in single-slot and double-slot bracket systems are scarce, and this trial aimed to address this gap by evaluating the effectiveness of the FFRD with both bracket systems. The null hypothesis assumed no significant differences in skeletal, dental, or soft tissue changes between the groups, allowing for a direct comparison of their clinical outcomes.

## Materials and methods

**Figure 1:**
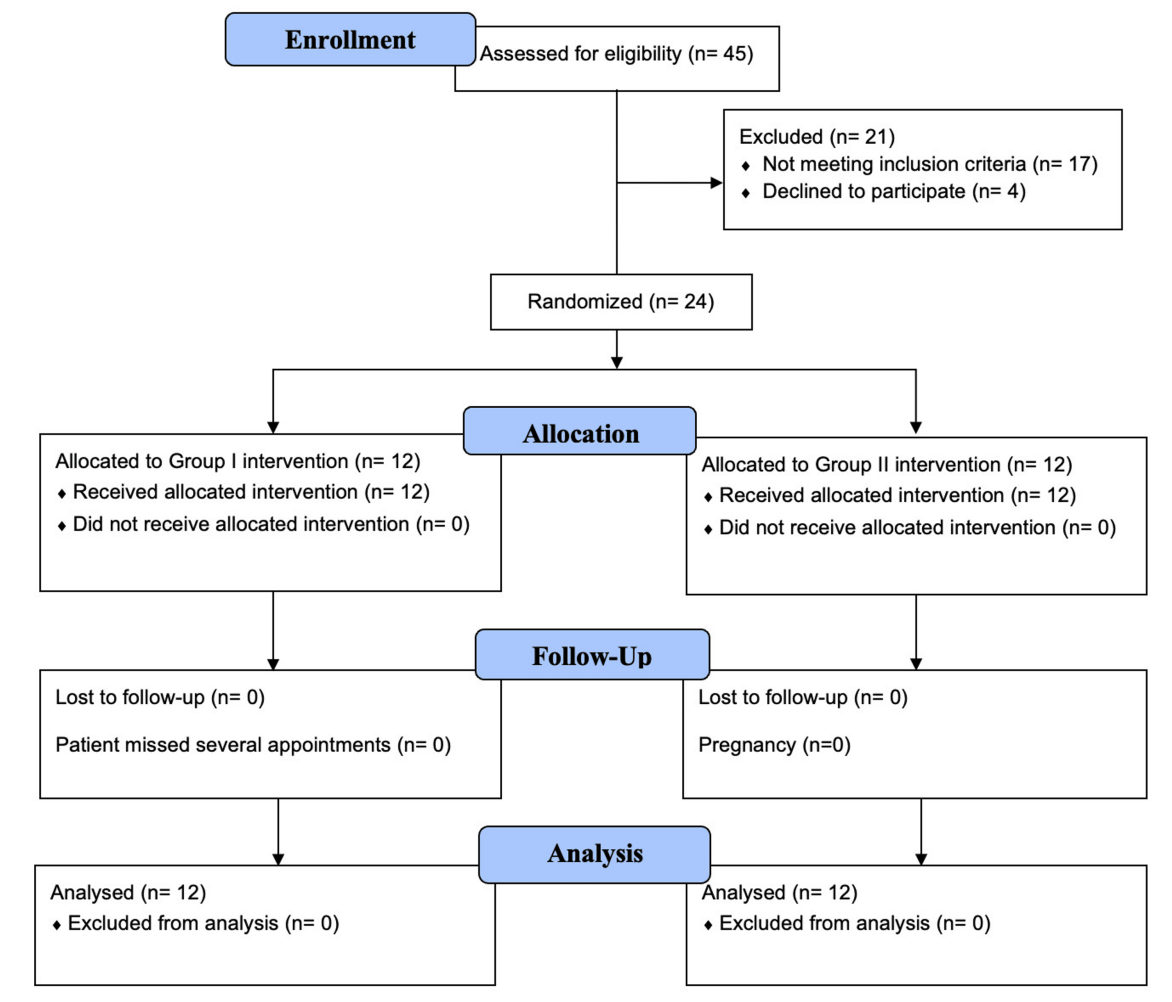
Consolidated Standards of Reporting Trials (CONSORT) flow diagram

A prospective randomized clinical trial was conducted in the Department of Orthodontics and Dentofacial Orthopedics at Banaras Hindu University, which was officially registered with the Clinical Trial Registry of India (CTRI) under registration number CTRI/2023/11/060368. The sample size was determined using G*Power software (version 3.1.9.7), with parameters set at a 5% Type I error rate, 80% power, and an effect size of 1.56, resulting in a sample size of 24 participants, with 12 allocated to each group. The study population comprised male and female patients aged between 15 and 20 years. This effect size was derived by substituting results from a previous study evaluating the effects of FFAs for the correction of Class II malocclusion. The high effect size is due to targeted nature of intervention, homogeneous sample selection, and standardized mechanics. Randomization was performed using a simple randomization technique to ensure a 1:1 allocation ratio. Allocation concealment was done using sequentially numbered, opaque, sealed envelopes prepared by an independent investigator. Blinding was implemented such that the participants and the data analyst were blinded, while the investigator blinding was not feasible due to the nature of the orthodontic intervention. The process utilized a chit-draw method for participant allocation. The included patients were circumpubertal individuals with Class II malocclusion Division 1, characterized by a retrognathic mandible, an overjet of 5-10mm, an average to horizontal growth pattern, a positive visual treatment objective, and minimal arch crowding. Participants with a history of prior orthodontic treatment, significant anterior tooth proclination or crowding, temporomandibular joint dysfunction, missing permanent teeth, facial asymmetry, parafunctional habits, or systemic conditions that could influence bone growth were excluded from the study. The patient flow throughout the study is illustrated in the Consolidated Standards of Reporting Trials (CONSORT) flow diagram (Figure 1).

**Figure 2:**
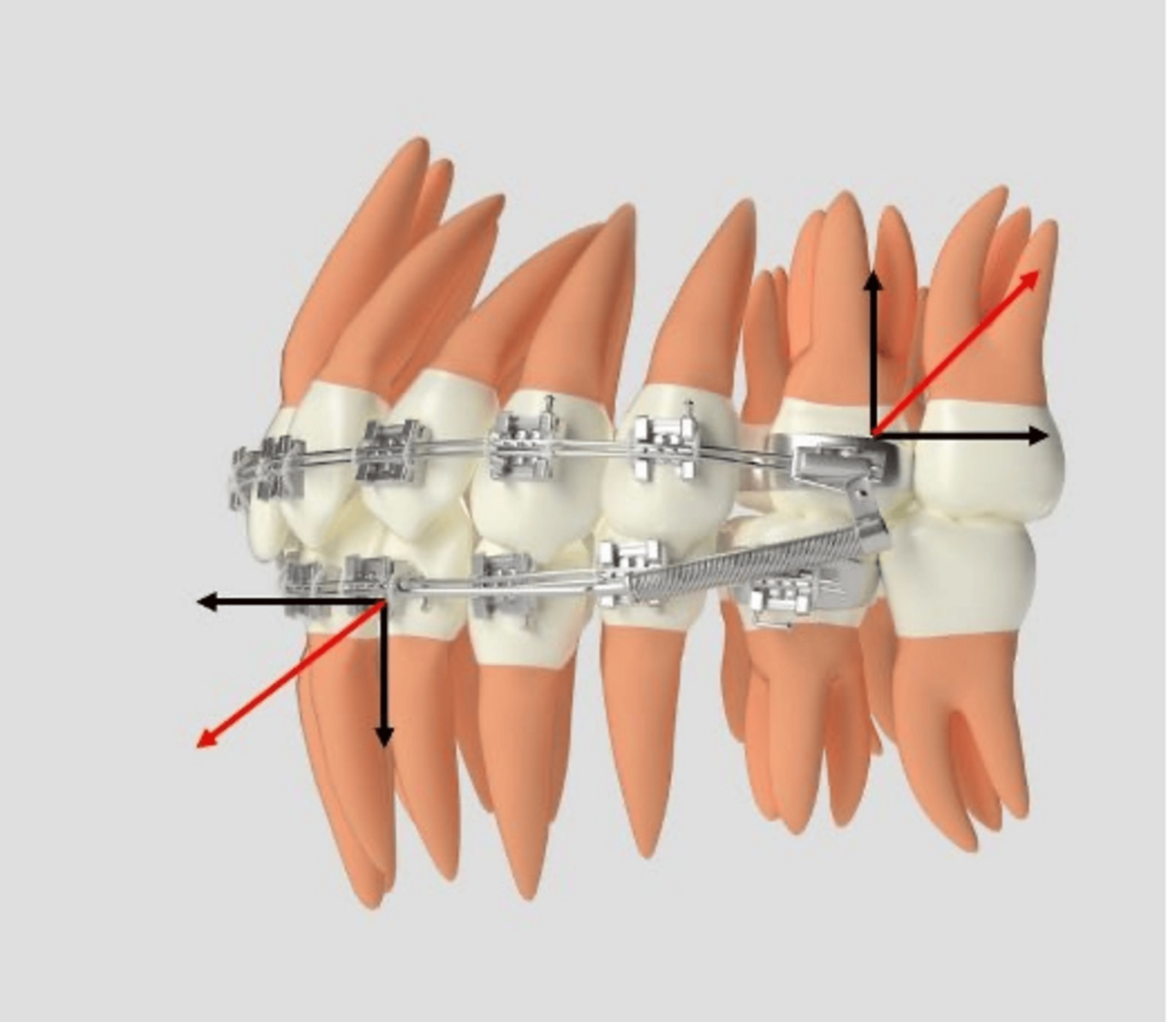
Forsus Fatigue Resistant Device Group 2 with double wire. Source: Self illustrated; No external sources.

**Figure 3:**
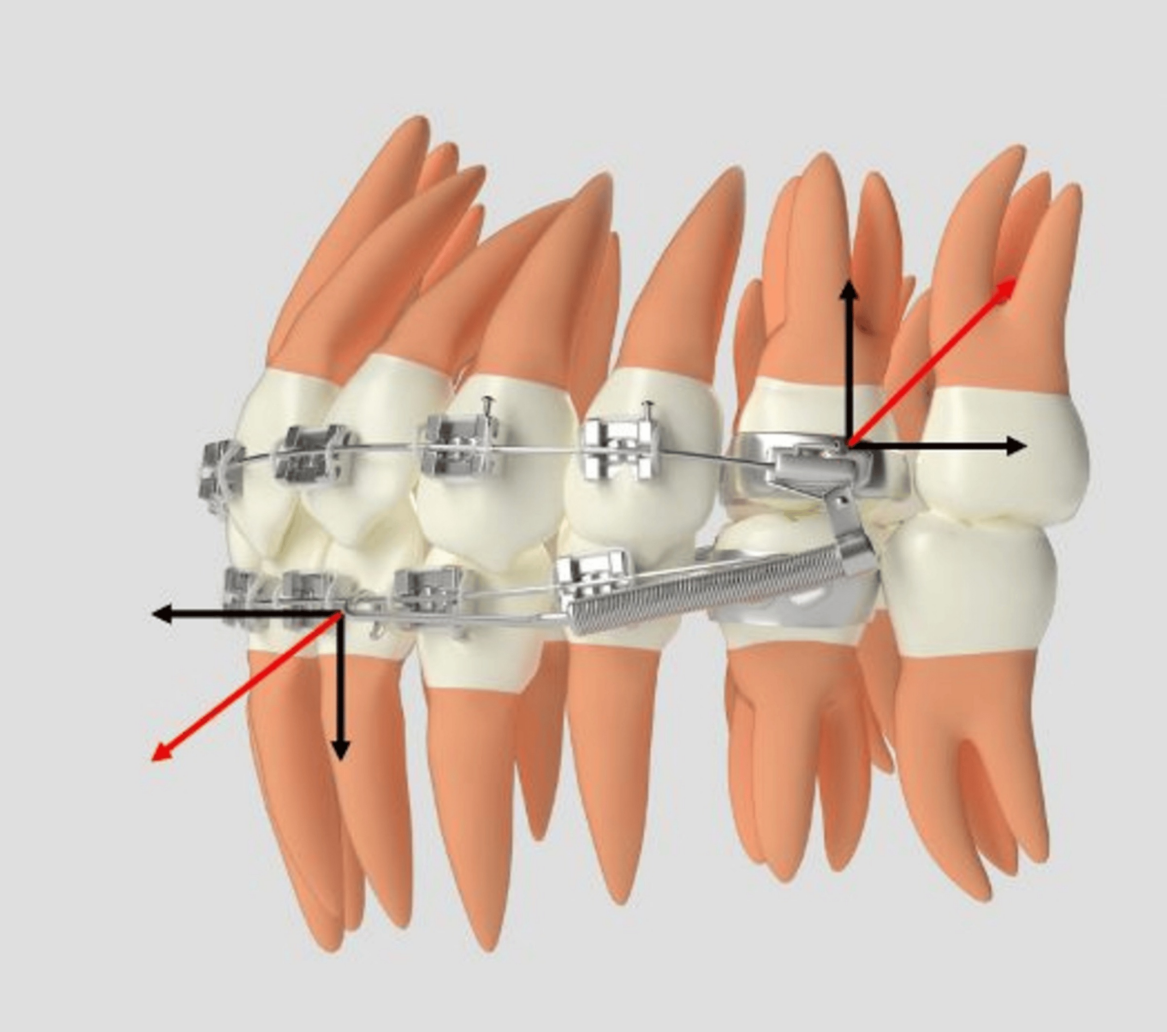
Forsus Fatigue Resistant Device Group 1 with single wire. Source: Self illustrated; No external source.

Written informed consent was obtained from all participants and their guardians after a detailed explanation of the study protocol. Twenty-four patients were randomly allocated to either Group 1, treated with single-slot brackets, or Group 2, treated with double-slot brackets according to the prescriptions outlined in Table 1. Group 1 utilized a single wire in single-slot brackets in conjunction with the FFRD, while Group 2 employed a double wire in double-slot brackets with the Forsus device, as seen in Figures 2, 3. Group 1 was treated using 0.022" × 0.028" brackets following the MBT prescription (Mini Diamond; Ormco Ltd., Brea, CA, USA), whereas Group 2 utilized dual-slot brackets with dimensions of 0.022" × 0.025" and 0.018" × 0.025" in Roth-prescribed appliances (Koden Sortech Duplo Slot; Kozhikode, India). Both groups commenced treatment with flexible nickel-titanium (NiTi) archwires. Subsequently, Group 1 progressed to 0.019" × 0.025" stainless steel (SS) wires, while Group 2 utilized both 0.019" × 0.025" SS wires in the 0.022-inch slot and 0.017" × 0.025" SS wires in the 0.018-inch slot, with auxiliary transpalatal and lingual arches for additional anchorage. After five to six months of levelling and alignment, the Forsus EZ2 appliance was installed bilaterally (Figures 2, 3). Patients were monitored every four to six weeks, with adjustments made as necessary. The functional appliance phase lasted six to eight months, during which the Forsus device remained in place until a Class I or super Class I molar and canine relationship was achieved. The majority of patients presented with an overjet 7-10mm; hence, reactivation of the appliance was performed at six to eight week intervals. Following the removal of the Forsus device, occlusal settling was facilitated using light intermaxillary elastics to achieve optimal results. Standardized lateral cephalograms were obtained at three distinct treatment stages: prior to bonding (T0), post-levelling and alignment (T1), and after the completion of functional appliance therapy (T2). Key skeletal, dentoalveolar, and soft tissue parameters relevant to the diagnosis were measured at each stage. The patients were blinded; however, the study design precluded blinding of the operator. To minimize bias, the data were de-identified and forwarded to an independent, blinded statistician for analysis. To assess intra-examiner reliability, 10 randomly selected cephalograms were retraced and remeasured by the same examiner at a two-week interval to reduce recall bias. Intra-examiner reliability was assessed using the intraclass correlation coefficient. 

**Table 1:** Prescription of Duploslot bracket (KODEN), and conventional bracket (MINI DIAMOND) CI, Central Incisor; LI, Lateral Incisor; C, Canine; PM, Premolar; M, Molar

	Torque°						
Maxillary	CI	LI	C	PM1	PM2	M1	M2
Group 1 MINI DIAMOND (Single slot)	17	10	-7	-7	-7	-14	-14
Group 2 KODEN (Duplo slot)	11	7	-2	-7	-7	0	0
Mandibular							
Group 1 MINI DIAMOND (Single slot)	-6	-6	-6	-12	-17	-20	10
Group 2 KODEN (Duplo slot)	0	0	-11	-17	-22	0	0
	Tip°						
Maxillary							
Group 1 MINI DIAMOND (Single slot)	4	8	8	0	0	0	0
Group 2 KODEN (Duplo slot)	5	8	9	0	0	0	0
Mandibular							
Group 1 MINI DIAMOND (Single slot)	0	0	3	2	2	0	0
Group 2 KODEN (Duplo slot)	0	0	5	0	0	0	0

Statistical analysis

Data were systematically recorded and analyzed using SPSS Statistics version 21 (IBM Corp., Armonk, NY, USA). A significance level of 5% (p < 0.05) was set for all statistical tests. Normality of the data was assessed using the Shapiro-Wilk test. For datasets not conforming to a normal distribution, non-parametric statistical methods were employed. The Mann-Whitney U test was utilized for inter-group comparisons, while the Friedman test was applied for analyzing intra-group variations over different time points. For parameters exhibiting a normal distribution, parametric tests were applied. Repeated measures ANOVA was used to evaluate intra-group changes across the three time points, and independent t-tests were conducted to compare inter-group differences. This dual statistical approach ensured the rigorous and appropriate analysis of both normally and non-normally distributed data.

## Results

Baseline comparisons of mean values and standard deviations at T0 indicated no statistically significant differences between the two groups.

Skeletal measurements

Intragroup analysis revealed a statistically significant increase in the SNB angle in both groups. Group 1 demonstrated a significant increase in the SNB angle from 77.75° ± 4.2° to 80° ± 4.72° (p < 0.001), while Group 2 exhibited an increase from 74.13° ± 3.68° to 75.88° ± 3.44° (p = 0.007). Similarly, Wits appraisal significantly decreased in both groups, from 4.25 ± 1.66 to 2.38 ± 1.76 in Group 1 (p = 0.006) and from 3.88 ± 1.12 to 0.5 ± 1.6 in Group 2 (p < 0.001). Frankfort-Mandibular plane angle (FMA) values also increased significantly in both groups, from 20.75° ± 5.99° to 24.5° ± 5.01° in Group 1 (p < 0.001) and from 25.50° ± 4.24° to 27.75° ± 4.77° in Group 2 (p = 0.002).

Intergroup comparisons using independent samples t-tests demonstrated no statistically significant differences in skeletal parameters at any time point. Specifically, Jarabak ratio did not differ significantly at T0 (t = 1.287, p = 0.219) or T1 (t = 1.510, p = 0.153); however, a statistically significant intergroup difference was observed at T2 (t = 2.411, df = 14, p = 0.032) and Wits appraisal at T2 (t = -1.571, df = 14, p = 0.048).

Dentoalveolar measurements 

The maxillary incisors showed improved inclination in both groups, with the U1-NA angle decreasing from 28.75° ± 3.69° to 24.5° ± 2.82° (p = 0.012) in Group 1 and from 24.25° ± 6.51° to 21.25° ± 5° (p < 0.001) in Group 2. Mandibular incisor proclination was observed in both groups; however, Group 2 demonstrated better control, with L1-NB angles changing from 32° ± 4.25° to 36.13° ± 3.56° (p = 0.297), compared to Group 1, where L1-NB angles increased from 29.88° ± 4.39° to 33.38° ± 3.37° (p = 0.065). Both groups exhibited a significant increase in incisor mandibular plane angle (IMPA) values, from 103.5° ± 2.97° to 109° ± 3.25° (p < 0.001) in Group 1 and from 106.25° ± 7.53° to 108.50° ± 7.17° (p = 0.012) in Group 2.

Intergroup comparisons revealed no statistically significant differences at T0, T1, or T2. Intergroup comparison using independent t-test demonstrated no statistically significant differences in IMPA at T0 (t = 0.599, df = 14, p = 0.565), T1 (t = −0.960, df = 9.132, p = 0.362), or T2 (t = 0.180, df = 9.761, p = 0.861).

Soft tissue measurements 

The nasolabial angle significantly increased in both groups, from 115.13° ± 7.24° to 118.5° ± 6.69° in Group 1 (p = 0.002) and from 105.38° ± 9.07° to 116.13° ± 8.13° in Group 2 (p = 0.001). Intergroup analysis of nasolabial angle revealed no significant difference at baseline (T0: t = 0.658, p = 0.521); however, a statistically significant intergroup difference was observed at T1 (t = 2.376, df = 14, p = 0.032). However, changes in upper and lower lip positions relative to the E-line were not statistically significant at any time point (upper lip T1: t = −1.273, p = 0.226; lower lip: p > 0.05).

**Figure 4:**
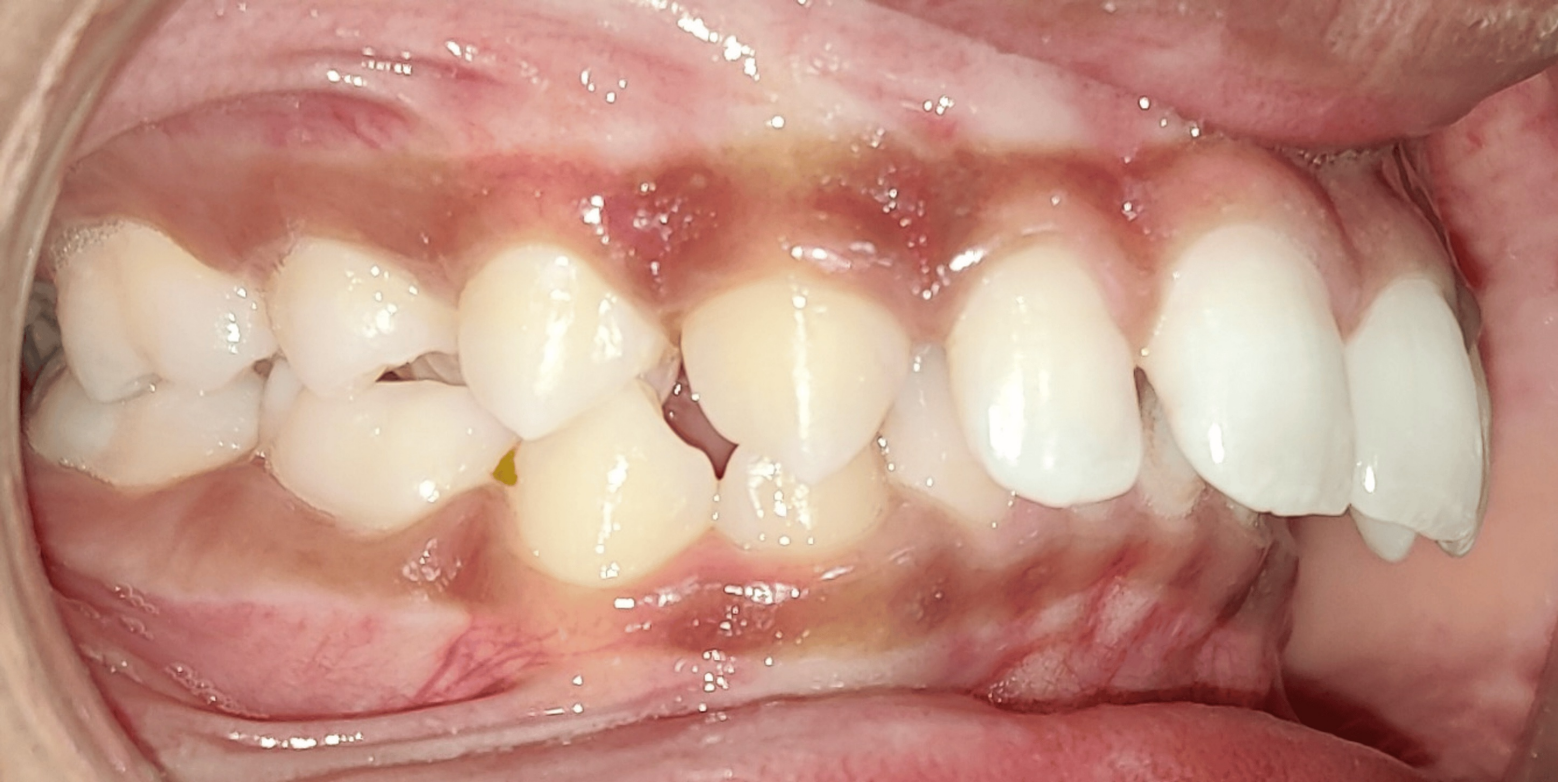
Pre-treatment.

**Figure 5:**
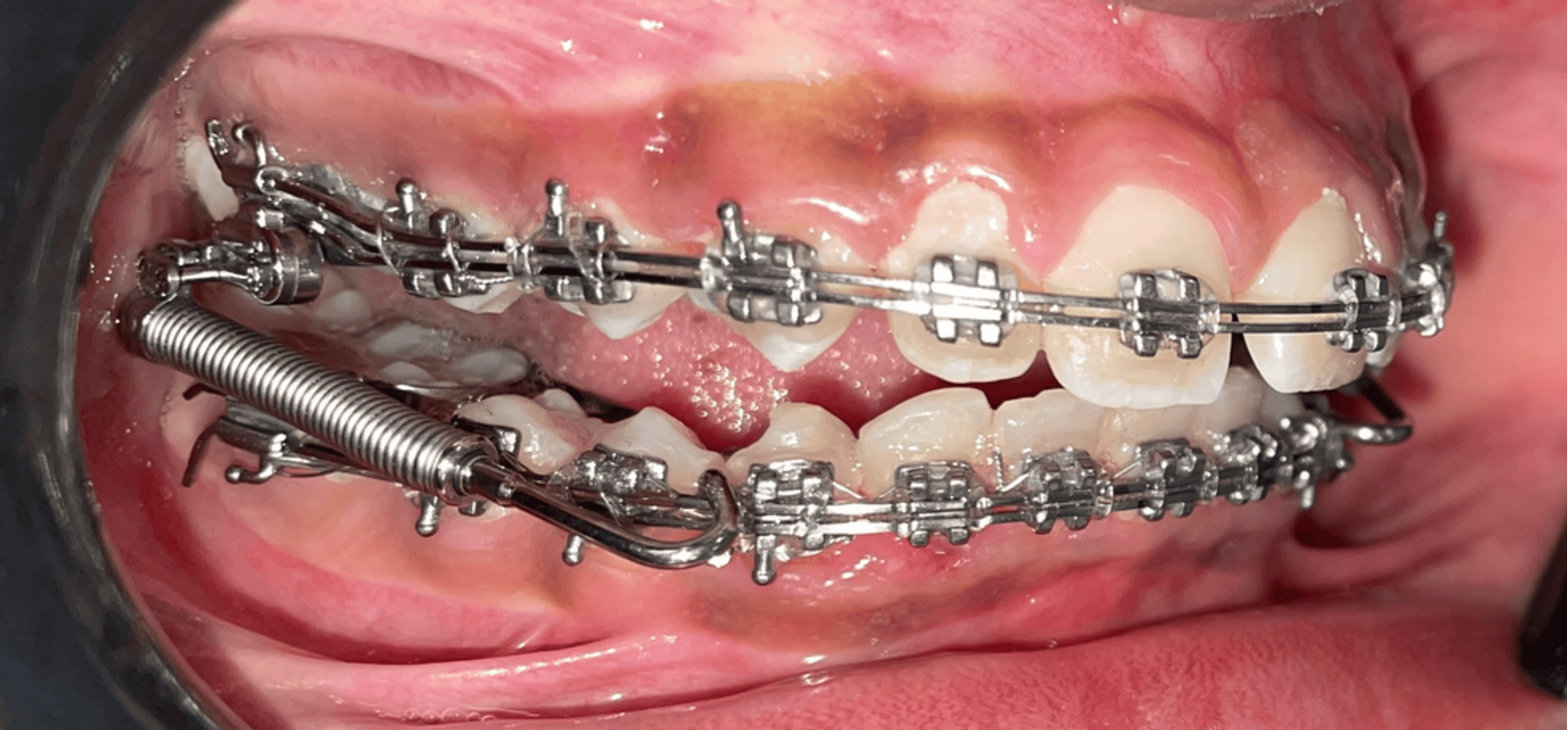
Forsus installation with double-slot bracket system.

Tables 2, 3, 4 provide data analysis for skeletal, dentoalveolar, and soft tissue measurements, respectively. Figures 4, 5 show pre-treatment stage and Forsus installation in the double-slot bracket system, respectively.

**Table 2:** Pre-treatment and post-treatment mean values, inter-group and intra-group p-values of skeletal parameters. T0, Pre-treatment; T1, Post-levelling and alignment; T2, Post-functional appliance therapy; FMA, Frankfort Mandibular Plane Angle. Significant at p<0.05; Data normality assessed via the Shapiro-Wilk test; Non-normally distributed data analyzed with the Mann-Whitney U test (inter-group) and Friedman test (intra-group); Normally distributed data analyzed with repeated measures ANOVA (intra-group) and independent t-tests (inter-group); effect size calculated using Cohen’s d.

Parameter	Group	T0	T1	T2	Intra-group p value
SNA (°)	Group 1	83.88 ± 4.94	84.38 ± 4.77	83.75 ± 5.06	0.373
	Group 2	80.50 ± 4.10	80.13 ± 4.12	79.63 ± 3.90	0.092
	Effect size	0.744	0.953	0.912	
	Inter-group p value	0.159	1.90	0.092	
SNB (°)	Group 1	77.38 ± 4.47	77.75 ± 4.20	80.00 ± 4.72	<0.001*
	Group 2	74.50 ± 3.20	74.13 ± 3.68	75.88 ± 3.44	0.007*
	Effect size	0.740	0.916	0.997	
	Inter-group p value	0.162	0.089	0.066	
ANB (°)	Group 1	6.75 ± 1.48	6.63 ± 1.76	3.88 ± 0.99	<0.001*
	Group 2	6.00 ± 1.60	6.00 ± 1.30	4.00 ± 1.40	<0.001*
	Effect size	0.486	0.407	0.098	
	Inter-group p value	0.362	0.435	0.842	
Wit’s appraisal (mm)	Group 1	4.75 ± 1.90	4.25 ± 1.66	2.38 ± 1.76	0.006*
	Group 2	3.88 ± 0.84	3.88 ± 1.12	0.50 ± 1.60	<0.001*
	Effect size	0.592	0.261	1.11	
	Inter-group p value	0.269	0.607	0.048*	
FMA (°)	Group 1	20.38 ± 5.70	20.75 ± 5.99	24.50 ± 5.01	<0.001*
	Group 2	24.63 ± 5.10	25.50 ± 4.24	27.75 ± 4.77	0.002*
	Effect size	0.786	0.915	0.664	
	Inter-group p value	0.138	0.089	0.205	
Jarabak ratio	Group 1	69.96 ± 4.80	70.03 ± 4.94	71.04 ± 4.82	0.125
	Group 2	66.71 ± 5.28	66.25 ± 5.05	55.97 ± 3.47	0.697
	Effect size	0.644	0.756	3.588	
	Inter-group p value	0.219	0.153	0.032*	

**Table 3:** Pre-treatment and post-treatment mean values, inter-group and intra-group p-values of dentoalveolar parameters. T0, Pre-treatment; T1, Post-levelling and alignment; T2, Post-functional appliance therapy; IMPA, incisor mandibular plane angle; U1, Upper central incisor; L1, Lower central incisor. Values are presented as mean + standard deviation. Significant at p<0.05; Data normality assessed via the Shapiro-Wilk test; Non-normally distributed data analyzed with the Mann-Whitney U test (inter-group) and Friedman test (intra-group); Normally distributed data analyzed with repeated measures ANOVA (intra-group) and independent t-tests (inter-group); effect size calculated using Cohen’s d.

Parameter	Group	T0	T1	T2	Intra-group p value
U1–NA (°)	Group 1 (Mean ± SD)	36.00 ± 8.86	28.75 ± 3.69	24.50 ± 2.82	0.012*
	Group 2 (Mean ± SD)	37.75 ± 7.45	24.25 ± 6.51	21.25 ± 5.00	<0.001*
	Effect size	0.213	0.850	0.800	
	Inter-group p value	0.765	0.111	0.132	
L1–NB (°)	Group 1 (Mean ± SD)	29.88 ± 4.39	30.88 ± 4.39	33.38 ± 3.37	0.065
	Group 2 (Mean ± SD)	32.00 ± 4.25	34.13 ± 4.45	36.13 ± 3.56	0.297
	Effect size	0.490	0.735	0.793	
	Inter-group p value	0.341	0.154	0.136	
IMPA (°)	Group 1 (Mean ± SD)	102.00 ± 2.56	103.50 ± 2.97	109.00 ± 3.25	<0.001*
	Group 2 (Mean ± SD)	100.25 ± 7.85	106.25 ± 7.54	108.50 ± 7.17	0.012*
	Effect size	0.299	0.480	0.080	
	Inter-group p value	0.565	0.362	0.861	
U1–NA (mm)	Group 1 (Mean ± SD)	6.75 ± 2.12	3.00 ± 0.92	2.25 ± 1.90	0.002*
	Group 2 (Mean ± SD)	7.88 ± 1.81	4.38 ± 1.85	3.88 ± 0.64	<0.001*
	Effect size	0.573	0.945	1.149	
	Inter-group p value	0.273	0.104	0.020*	
L1–NB (mm)	Group 1 (Mean ± SD)	3.75 ± 0.71	3.50 ± 1.06	4.75 ± 3.18	0.075
	Group 2 (Mean ± SD)	3.63 ± 2.56	4.38 ± 1.85	5.88 ± 1.46	0.037*
	Effect size	0.063	0.584	0.456	
	Inter-group p value	0.897	0.050*	0.136	

**Table 4:** Pre-treatment and post-treatment mean values, inter-group and intra-group p-values of soft-tissue parameters. T0: Pre-treatment; T1, Post-levelling and alignment; T2, Post-functional appliance therapy; Values are presented as mean ± standard deviation. Significant at p<0.05; Data normality assessed via the Shapiro-Wilk test; Non-normally distributed data analyzed with the Mann-Whitney U test (inter-group) and Friedman test (intra-group); Normally distributed data analyzed with repeated measures ANOVA (intra-group) and independent t-tests (inter-group); effect size calculated using Cohen’s d.

Parameter	Group	T0	T1	T2	Intra-group p value
Nasolabial angle (°)	Group 1	106.63 ± 8.48	115.13 ± 7.24	118.50 ± 6.69	<0.001*
	Group 2	103.75 ± 8.99	105.38 ± 9.07	116.13 ± 8.13	<0.001*
	Effect size	0.329	1.188	0.318	
	Inter-group p value	0.521	0.330	0.367	
Upper lip to E-line (mm)	Group 1	0.63 ± 2.44	−0.75 ± 2.49	0.13 ± 2.99	0.193
	Group 2	0.88 ± 2.03	0.63 ± 1.77	−0.25 ± 1.28	0.110
	Effect size	0.111	0.639	0.165	
	Inter-group p value	0.827	0.226	0.226	
Lower lip to E-line (mm)	Group 1	−0.13 ± 2.94	−0.38 ± 2.72	0.88 ± 2.29	0.386
	Group 2	0.63 ± 1.85	1.38 ± 2.26	2.38 ± 1.85	0.015*
	Effect size	0.309	0.703	0.721	
	Inter-group p value	0.554	0.185	0.173	

## Discussion

The Forsus is a popular, non-compliance-based appliance for the correction of Class II malocclusion. While it offers a relatively short active treatment time of about six months, its specific skeletal and dental effects can vary between studies [13]. The mesial movement of the mandible observed in the single-slot group aligns with prior studies on Forsus appliances [11,14-21]. Limited data exists on double-slot bracket systems [15]. Both groups demonstrated significant mandibular advancement (SNB; p < 0.05), with no statistically significant differences between them, indicating similar skeletal efficacy. To the author's knowledge, this study is the first of its kind to evaluate the clinical outcomes of FFAs when utilized with both single and double-slot bracket systems. Maxillary skeletal changes showed no significant differences between groups, consistent with existing research [9,22]. Both groups exhibited a significant reduction in the ANB angle due to sagittal adjustments in both jaws [23,24]. Apical base changes (Wits appraisal), reflecting maxillomandibular differentials, showed significant overall differences (p < 0.05), but these were not statistically significant between groups. Mandibular plane angle increased in both groups, with vertical changes such as Jarabak’s ratio reflecting the downward and forward forces applied by the appliances. These findings are consistent with studies reporting vertical condylar growth and alterations in mandibular position induced by FFA therapy [18,25,26].

Both groups demonstrated distal movement of the maxillary dentition and mesialization of the mandibular molars, alongside proclination of the lower incisors. Mean overjet decreased in both groups over the observation period. In Group 1, overjet decreased from 8.88±1.64mm at baseline to 2.75±1.03mm following FFA therapy, while Group 2 showed a reduction from 9.50±1.03mm at baseline to 2.25±0.71mm at the end of treatment, paralleling outcomes in previous FFA studies [1,18,27,28]. This suggests that dual wires in double-slot brackets did not markedly influence the dentoalveolar effects of the Forsus appliance. The study highlighted that the rigidity of dual wires and reduced play in the 0.018-inch slot of Duplo-slot brackets might have contributed to better control of lower incisor proclination in Group 2, despite the Roth prescription used for these brackets. Other methods, such as skeletal anchorage with miniscrews [11,18,29,30] or miniplates [29,30], brackets with enhanced negative torque [29,30], or systems like the Butterfly Bracket System [29], have shown promise in mitigating incisor proclination during FFA therapy. Both groups exhibited a significant increase in the nasolabial angle and improvements in upper and lower lip positions [30]. These findings generally align with previous studies, although some studies report insignificant changes with respect to lower lip position [9,11].

Limitations and future recommendations

While a consistent treatment protocol was rigorously adhered to in both groups, the relatively small sample size limits the ability to draw definitive conclusions about the comparative efficacy of single-slot and double-slot brackets in FFA therapy. Although both groups followed the same treatment framework, the distinct bracket designs and varying prescriptions between the two groups may have contributed to differences in treatment outcomes. The potential advantages of double-slot brackets warrant further investigation. Future studies could explore integrating skeletal anchorage or advanced bracket designs to enhance treatment outcomes and minimize undesirable side effects.

## Conclusions

Both single-slot and double-slot groups exhibited significant improvements in all measured parameters, including mandibular advancement, overjet reduction, and facial aesthetics. However, intergroup comparisons revealed no statistically significant differences in skeletal, dental, or soft tissue outcomes. These findings support the null hypothesis, suggesting that both single wires and double wires are equally effective with the FFRD in managing mandibular retrusion.

On the clinical bases, the data implies that the choice between single and double wire mechanics is independent of treatment outcomes and purely based on patient comfort, appliance simplicity, cost, and adjunctive treatment mechanics such as torque control or space closure. Therefore, clinicians can adopt either wire configuration while expecting similar therapeutic efficiency when treating skeletal Class II malocclusion characterized by mandibular retrusion using the FFRD.
